# Edward Hugh Edwards Stack

**Published:** 1922-09

**Authors:** J. A. Nixon


### Obituary.

### EDWARD HUGH EDWARDS STACK,
### M.B., B.Ch. Cantab., F.R.C.S. Eng.

THE death of Mr. Stack at the early age of 55 came as a great shock to his friends. He had been in London undergoing treatment for an internal neoplasm. His letters were full of hope, and he had written to say that he would start work again in September. Then without further warning we heard he had died on August 3rd. He had suffered bravely for many months the pain due to secondary deposits of hypernephroma in the bones.

Mr. Stack was born at Langfield, County Tyrone, in 1866, the third son of the Rev. Canon Stack. He was educated at Haileybury, Pembroke College, Cambridge, and at St. Bartholomew's Hospital. As a medical student he was noted for working hard and for playing games hard too. Although he did not excel in any branch of athletics, he represented his hospital in nearly every sport at one time or another.

Dr. J. A. Arkwright, a fellow-student of his at Bart's, writes :—

"I have the most delightful recollection of E. H. E. Stack as an intensely keen student and as Dr. Gee's house physician at Bart's. His enthusiasm and single-minded pursuit of all that pertained to the knowledge of medicine, and especially clinical medicine (including surgery) for its own sake, stand out very clearly in my mind. He had considerable mechanical skill and ingenuity, and always preferred making things himself to accepting ready-made apparatus. This love of handicraft and personal contact with the details of his profession gave a freshness and originality to his work and outlook which were very inspiring.

"We were almost contemporaries, and I remember well his untiring energy in studying and following up cases that he had seen and noted. His zeal in inquiring into their diseases was joined to quite an unusual degree of interest in the patients from the standpoint of humanity. Unfortunately, we have seldom met or exchanged letters since those days ending in 1893, but I have continued to regard him as an always reliable friend. He was exceptionally gifted and an honest man, who continued through life a serious student of the profession which he loved. I was much saddened by hearing of his death at an age when his ripe experience and great fund of accumulated first-hand knowledge would have been of untold value to the rising generations of students and to those patients whom he was called upon to advise and treat."

This description of Stack in his student days will hold good of him to the end. As a resident at the Royal Infirmary we can recall the same keen insight into the personalities as well as the ailments of his patients, and his memory never failed him either about their symptoms or their family circumstances. His love of the mechanical arts and crafts he retained throughout life. Whilst he was House Surgeon at the Bristol Royal Infirmary if Stack was ever wanted the first place to look for him was the engineer's or the carpenter's shop, last of all his own room.

Stack's interest in and enthusiasm for the welfare of the students will never be forgotten. He helped to start *The Stethoscope,* the journal of the Bristol Medical School, which has flourished for more than twenty years and shows no signs of lessened vitality. For many years Stack managed a valuable department of clinical pathology in connection with *The Stethoscope* at a time when the practitioners in Bristol and the West of England were beginning to realise the need of such a department. The work he did was appreciated far and wide. He launched the movement that amalgamated the medical athletic clubs into a single union long before the University came into existence. Year by year the success of the Bristol Medical Dinner bore witness to his organising energies. The Medical Dramatic Club owed much to his ability in many capacities, either as actor, stage carpenter, or scene painter.

This reminds us that Stack possessed great artistic talents and much skill in the handicrafts, and it was these gifts which made him the sound clinician that he undoubtedly was. Few men were less puzzled by cases presenting uncommon features. Sometimes his intuition seemed to be mere happy guessing, but his acute perception enabled him to appreciate signs and symptoms that would escape a less exact observer, so that when he "guessed" the dice were usually loaded in his favour by something he had noticed. To students his "grinds" and ward classes were always interesting, and his teaching was imparted in a form not easy to forget.

He held many medical appointments, first of all as a resident at Bart's and afterwards in Bristol. In 1906 he was elected Assistant Surgeon at the Royal Infirmary. In 1914 he became full Surgeon, but on Dr. Ogilvy's death in that year he decided to specialise in ophthalmology, and succeeded to the vacant post of ophthalmic surgeon. He was also Surgeon to the Bristol Eye Hospital, and had held appointments at the Cossham and the Orthopædic Hospitals.

The South-Western Ophthalmological Society was founded by him in 1920.

Stack was not a prolific writer, but he had published a few articles indicative of an observant mind and dexterous hand.

Stack was a staunch Churchman, and at the time of his death filled the office of Churchwarden at St. Paul's, Clifton.

A type of the warm-hearted, energetic, loyal Ulsterman, and generous and staunch to friends, he was a man without an enemy and beloved by his colleagues. But to know Stack one could not do better than take part in the social re-unions he organised for students and young practitioners, or be one at his family gatherings with his wife and children around, when all shared with the father the duty and pleasure of dispensing kindly greetings and hospitality. His widow and four young children are not alone in mourning the death of this good man, who was held in such esteem by all those amongst whom he lived and worked.

J. A. NIXON.

BIBLIOGRAPHY.

"Notes on Hepatic Cirrhosis in Children," *Practitioner*, 1892, xlviii. 186–193.

"Specialism in General Practice," *St. Barth. Hosp. J.*, 1897–8, v. 7.

"A Case of Diffuse Leontiasis Ossea," *Bristol M.-Chir. J.*, 1900, xviii. 316-320.

"Six Cases of Cerebro-Spinal Meningitis," *Ibid.*, 1901, xix. 44–50.

(With E. W. H. Groves.) "A New Operating Table," *Lancet*, 1908, ii. 1141–1143.

"A Family with a Congenital Deformity of the Nose, and the results of Subcutaneous Injection of Wax," *Bristol M.-Chir. J.*, 1909, xxvii, 119–124.

"Some Gall-bladder Cases presenting Features of Interest," *Ibid.*, 1912. xxx. 49–52.

"Gunshot Injuries to the Eyes," *Ibid.*, 1915, xxxiii. 198–206.

(With Drs. Coombs and Rolfe.) "Poisoning by 'Mustard' Gas," *Ibid.*, 1920, xxxvii. 151.

"Intubation in Diphtheria," Allbutt and Rolleston's *System of Medicine*, 1905, i. 1023.

